# Supplementary material for: ECM Substrates Impact RNAi Localization at Adherens Junctions of Colon Epithelial Cells
Source: Cells. 2022 Nov 23;11(23):3740. doi: 10.3390/cells11233740 (PMC9737857; doi:10.3390/cells11233740)
Supplement: Supplementary file 1 [file cells-11-03740-s001.zip › cells-1922696-supplementary.pdf]

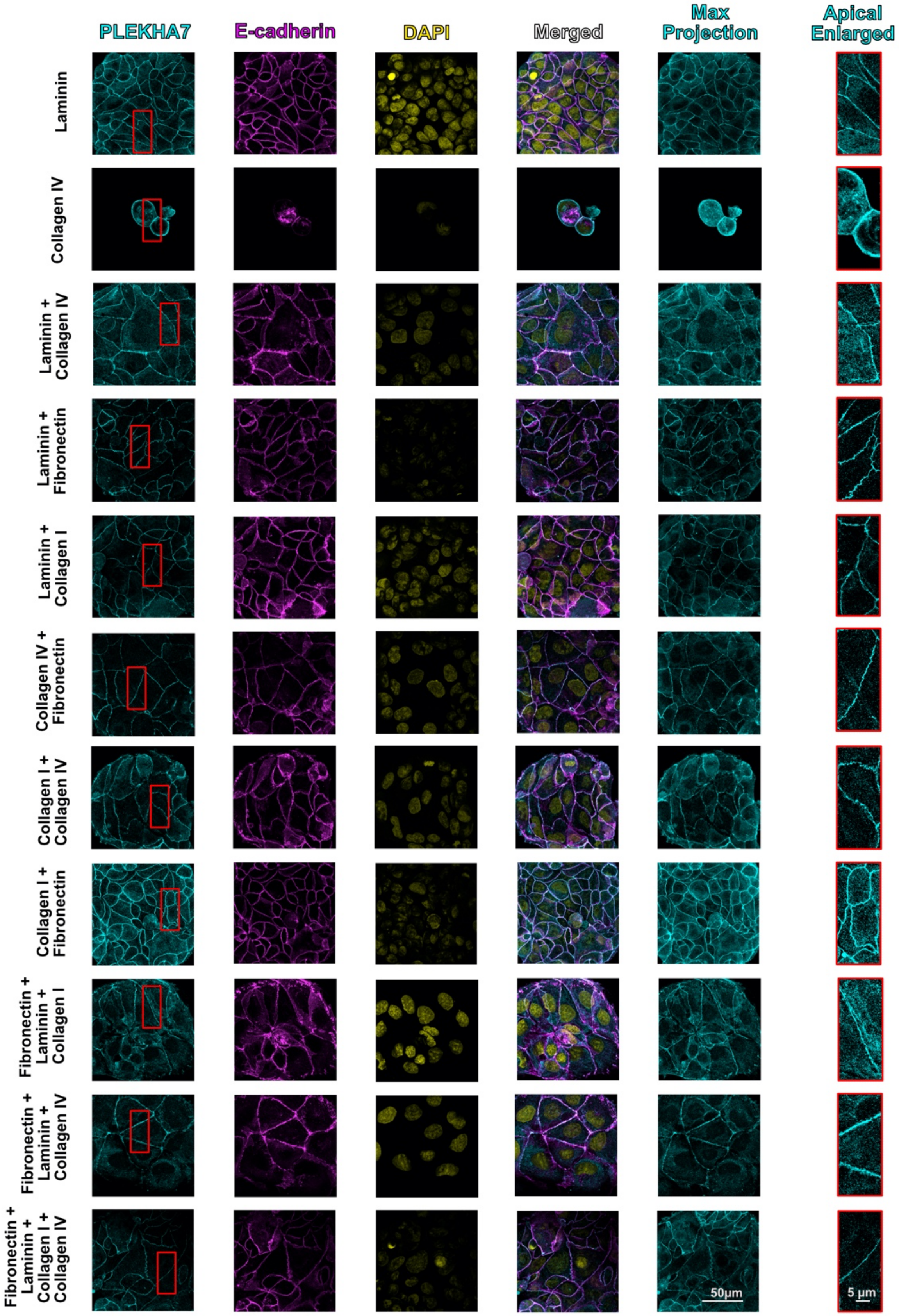

Figure S1. Representative immunofluorescence images of Caco2 colon epithelial cells, plated on different combinations of ECM proteins, stained for PLEKHA7 (magenta), E-cadherin (cyan), and nuclei (DAPI; yellow).

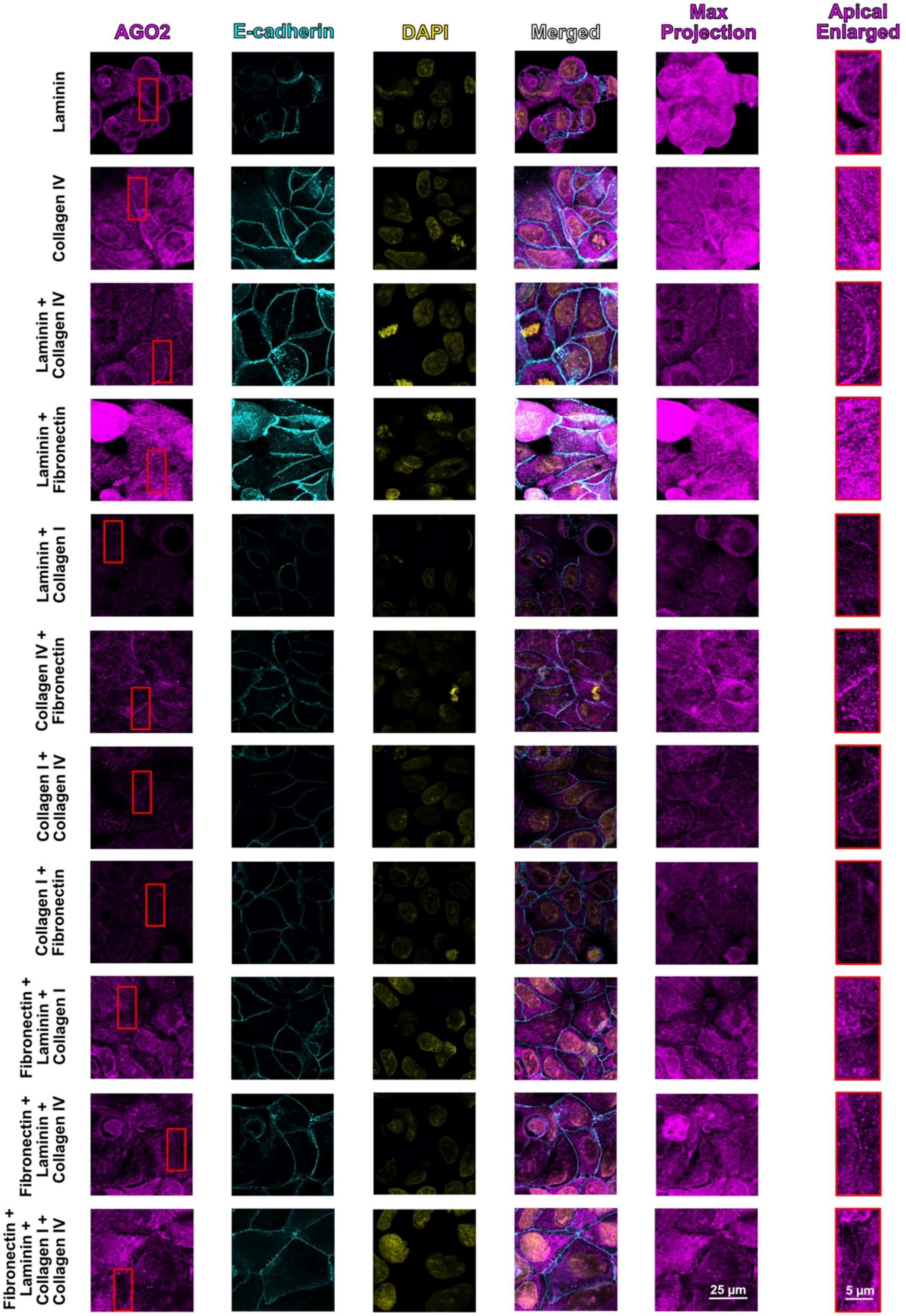

Figure S2. Representative immunofluorescence images of Caco2 colon epithelial cells plated on different combinations of ECM proteins stained for AGO2 (magenta), E-cadherin (cyan), and nuclei (DAPI; yellow).

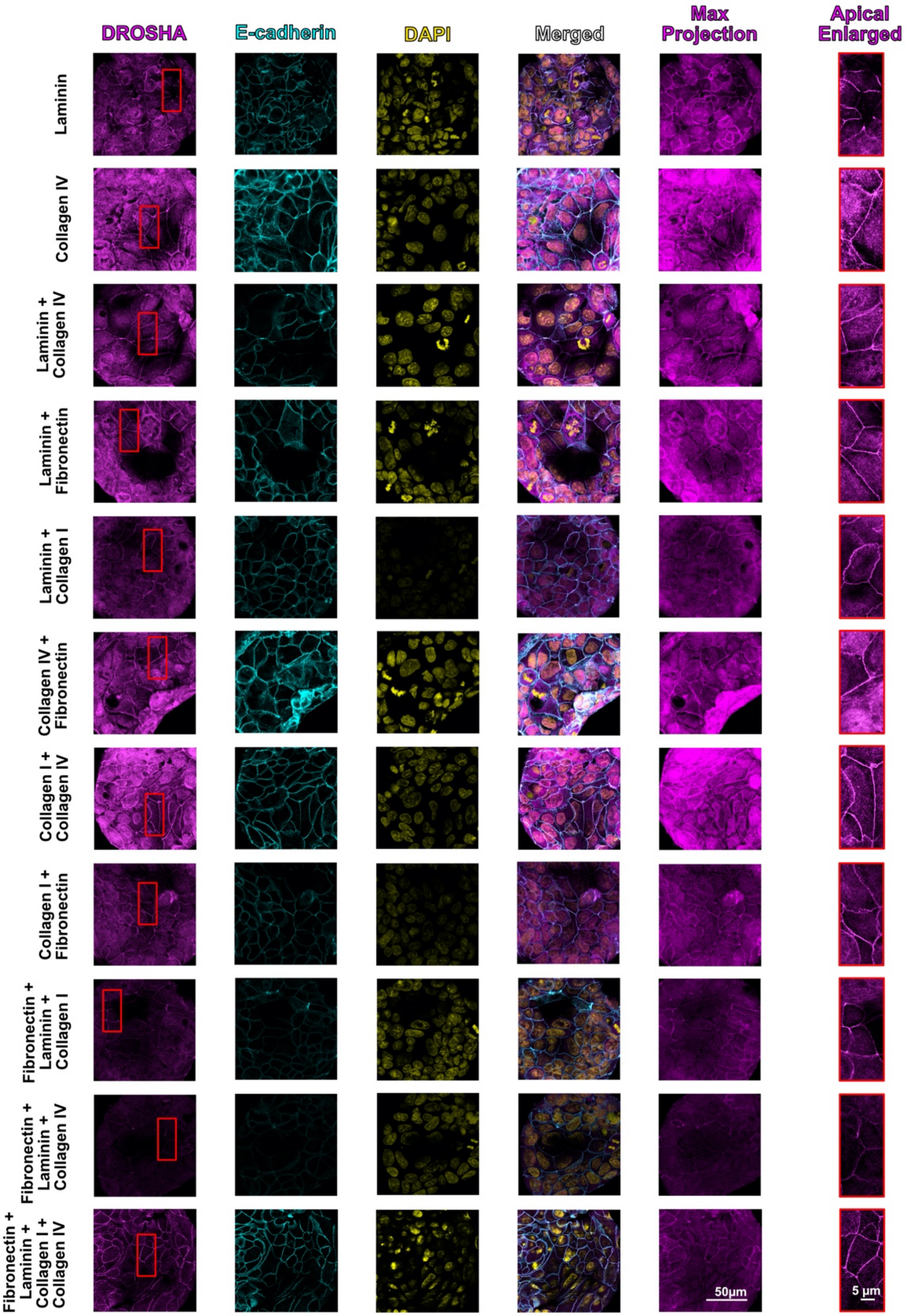

Figure S3. Representative immunofluorescence images of Caco2 colon epithelial cells plated on different combinations of ECM proteins stained for DROSHA (magenta), E-cadherin (cyan), and nuclei (DAPI; yellow).
